# Nanoparticles in Vascular Dementia: Advantages and Challenges

**DOI:** 10.1002/cns.70966

**Published:** 2026-06-04

**Authors:** Xianxin Liu, JiaLi Hu, Yueming qiu, Huaying Wang, Chunxiao Shen, Ziying Liu, Yiting He, Xuefei Du, Xiaorong Zhang

**Affiliations:** ^1^ Department of Pathology Clinical Medical School of Jiujiang University Jiujiang Jiangxi China; ^2^ School of Materials Science and Engineering Jiujiang University Jiujiang Jiangxi China; ^3^ Jiujiang Clinical Precision Medicine Research Center Jiujiang Jiangxi China; ^4^ Department of Pathology Affiliated Hospital of Jiujiang University Jiujiang Jiangxi China; ^5^ Jiangxi Province Engineering Research Center of Materials Surface Enhancing & Remanufacturing Jiujiang University Jiujiang Jiangxi China

**Keywords:** biocompatibility, blood–brain barrier (BBB), drug‐release systems, nanomaterials, nanotechnology, neuroinflammation, vascular dementia (VaD)

## Abstract

**Background:**

Vascular dementia (VaD) is the second most prevalent form of dementia, accounting for 15%–20% of dementia cases and is primarily caused by cerebrovascular disorders. Current treatments predominantly focus on risk factor management and symptom alleviation, lacking the ability to cure the disease or reverse cognitive decline, which underscores the urgent need for novel therapeutic approaches like nanotechnology.

**Methods:**

This paper provides a comprehensive review integrating materials science, clinical medicine, and bioinformatics to evaluate the current status of nanomaterial research in VaD. It focuses on the molecular mechanisms of action of various nanomaterials and provides an analysis of their therapeutic potential and future development challenges.

**Results:**

In diagnostic applications, nanomaterials (e.g., SPIONs and fluorescent nanoparticles) enable high‐contrast imaging of early‐stage vascular inflammation, blood–brain barrier (BBB) leakage, and amyloid β (Aβ) deposits. In therapeutic applications, engineered nanocarriers (including liposomes, polymeric nanoparticles, and gold nanoparticles) exhibit specific targeting, controlled release, and BBB penetration capabilities. They mitigate VaD pathology by promoting angiogenesis, scavenging reactive oxygen species (ROS) to reduce oxidative stress, inhibiting neuroinflammation, protecting the neurovascular unit (NVU), and facilitating white matter repair.

**Conclusion:**

Nanotechnology provides an innovative framework for the diagnosis and treatment of VaD through intelligent, targeted drug delivery systems. Future advancements require establishing systematic biocompatibility evaluation frameworks, addressing long‐term toxicity concerns, and expanding multicenter clinical trials to validate safety and efficacy for clinical translation.

## Graphical abstract

This review highlights the dual diagnostic and therapeutic potential of nanomaterials in Vascular Dementia (VaD). By effectively crossing the blood–brain barrier, functionalized nanoparticles facilitate early diagnosis by targeting Aβ deposits and vascular lesions. Therapeutically, they ameliorate vascular injury, inhibit neurotoxic aggregation, and reduce both neuroinflammation and neuronal loss, ultimately protecting the neurovascular unit.

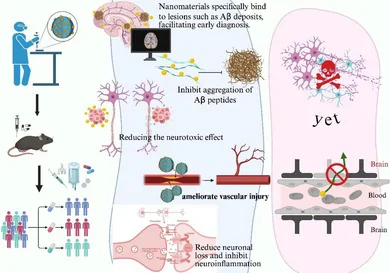

## 1 Introduction

Vascular dementia (VaD) is a neurocognitive disorder characterized by severe cognitive impairment caused by cerebrovascular diseases. It is associated with a reduction in cerebral blood flow, leading to neuronal damage and a decline in cognitive abilities [1]. VaD affects the quality of life of millions of people worldwide and imposes a heavy burden on families and societies [2, 3, 4, 5]. With the intensification of population aging, the incidence and prevalence of VaD are expected to continue rising, making the need for effective treatment methods particularly urgent [6]. Despite a growing understanding of VaD, current treatment methods mainly focus on controlling risk factors and alleviating symptoms. However, there is still no proven way to cure the disease or reverse the associated cognitive decline [6]. The treatment dilemma mainly stems from the complex pathophysiological mechanisms of VaD, which include changes in cerebral hemodynamics, neuronal damage, inflammatory responses, and disruption of the blood–brain barrier (BBB) [7].

Nanomaterials exhibit great potential in the treatment of VaD. Graphene quantum dots can penetrate the BBB, inhibit the fibrillation of α‐synuclein, depolymerize mature fibrils to reduce their harmful effects, improve mitochondrial function, and rescue neurons and synapses [8, 9]. After being modified and functionalized, metal nanoparticles (NPs) can cross the BBB to exert therapeutic effects [10, 11, 12]. Dendrimers play a crucial role in drug delivery, targeted therapy, and imaging technology due to their unique properties [13, 14, 15, 16, 17, 18, 19]. Carbon nanotubes can serve as targeted drug carriers, showing great promise for the treatment of central nervous system (CNS) diseases [20, 21, 22, 23, 24]. Polymeric NPs and liposomes are important components of drug delivery systems [25]. Liposomes can cross the BBB through various mechanisms [26, 27, 28, 29, 30], and their efficiency and safety can be enhanced through optimization [31]. In terms of diagnosis, nanomaterials such as polyethylene glycol superparamagnetic iron oxide NPs (SPIONs) combined with antibodies, as well as fluorescent NPs conjugated with specific markers, can detect and locate the deposition of amyloid β protein (Aβ) in cerebral blood vessels, supporting early diagnosis and treatment of VaD [32, 33, 34]. All these aspects comprehensively demonstrate the great potential of nanomaterials in the treatment of VaD [24, 35, 36, 37, 38, 39, 40].

This article reviews the interdisciplinary use of nanomaterials in VaD treatment, integrating materials science, bioinformatics, and clinical medicine. It highlights the need for intelligent drug‐release systems based on the pathological microenvironment for precise treatment. It also explores nanomachine's preventive potential in chronic inflammation and VaD risk reduction via early vascular intervention and advocates a global safety database and ethical standards for nanomaterials to facilitate clinical translation.

## 2 Overview of VaD

### 2.1 Epidemiology and Risk Factors

The incidence of VaD, a common type of dementia that affects the elderly population worldwide, varies across different regions and studies. VaD accounts for approximately 15% to 20% of all dementia cases and is particularly prevalent in North America and Europe [3, 41], where gender differences have been observed in its prevalence rate. Among individuals aged 65 and above, there is no significant difference in the cumulative risk of VaD between men and women. However, women are twice as likely as men to develop VaD in their later years [42]. Nevertheless, in Asia and some developing countries, this proportion may be even higher, reaching around 30% [43].

To understand the etiology of VaD, it is essential to distinguish between direct causes, systemic risk factors, and underlying pathophysiological mechanisms.

Primary Causes: The direct causes of VaD include cerebral infarction (stroke), small vessel diseases (SVD), vascular malformations, and specific blood disorders. Genetic conditions affecting cerebral microvasculature, such as CADASIL [44] and CARASIL [45], also serve as direct drivers of the disease.

Risk Factors: Systemic conditions such as hypertension, diabetes mellitus, hyperlipidemia, and heart diseases significantly increase the susceptibility to vascular damage. Additionally, unhealthy lifestyle factors, including smoking and excessive alcohol consumption, are critical modifiable risk factors [46, 47, 48, 49, 50, 51, 52].

Pathophysiological Mechanisms: These causes and risk factors trigger complex biological cascades, including chronic hypoperfusion, endothelial dysfunction, disruption of the blood–brain barrier (BBB), oxidative stress, and neurovascular inflammation. These mechanisms collectively lead to neuronal loss and the characteristic decline in cognitive function.

### 2.2 Diagnosis

Formal diagnostic criteria for VaD emerged in the 1980s–1990s [53, 54], evolving into standardized tools like the Hachinski Ischemic Scale [55] and the ADDTC [56] and NINDS‐AIREN criteria [57]. While diagnostic frameworks for vascular cognitive impairment remain debated [58], three core elements persist: (1) confirmed cognitive decline; (2) evidence of cerebrovascular damage; and (3) a causal link between them [59]. VaD diagnosis hence integrates clinical symptoms, neuroimaging, and vascular etiology.

### 2.3 Pathophysiology

VaD is closely intertwined with the pathological mechanisms of cerebrovascular diseases. Ischemia, brain tissue necrosis, and inflammatory responses triggered by cerebrovascular diseases are key factors in VaD development [60]. Its pathological mechanisms mainly involve small vessel disease (SVD) and large vessel disease (LVD) [2]. Imaging features of SVD include lacunes, white matter lesions, cerebral microbleeds, and enlarged perivascular spaces. Caused by acute ischemia and/or chronic hypoperfusion, these lesions lead to endothelial dysfunction, disruption of the BBB, oxidative stress, inflammation, and coagulation pathway disorder, ultimately resulting in neuron and glial cell degeneration [61, 62].

The disruption of the BBB is a crucial factor in SVD. The BBB is an integral part of the neurovascular unit (NVU), which includes endothelial cells, pericytes, smooth muscle cells, neurons, astrocytes, and microglia [63]. Multiple factors such as endothelial dysfunction and pericyte degeneration can lead to the disruption of the BBB [64, 65]. The latter causes substances in the blood vessels, such as fibrinogen, to exude, activating microglia and astrocytes, which produce reactive oxygen species (ROS) leading to oxidative stress [66].

Oxidative stress and inflammatory responses are major contributors to SVD. Microglia activation leads to the generation of pro‐inflammatory factors such as tumor necrosis factor alpha (TNF‐α) and interleukins (IL‐1, IL‐6, IL‐8), which participate in neuroinflammation and neuronal damage [67]. These pathological mechanisms work together to impair cerebral blood flow and damage brain tissue, ultimately affecting cognitive function and shaping the clinical characteristics of VaD. Understanding these mechanisms is therefore crucial for the development of targeted prevention and treatment strategies to improve the prognosis of patients with VaD.

## 3 Overview of NPs and Research Status of Nanomaterials in Medicine

Nanomedicine, an interdisciplinary field integrating nanotechnology and medicine, has significantly transformed the diagnosis and treatment of diseases since its practical application began in the 1990s. Its theoretical foundation can be traced back to the pioneering concept of manipulating matter at the molecular scale, proposed by physicist Richard Feynman in 1959 [68]. This field has achieved breakthroughs in the treatment of nervous system diseases, including overcoming the BBB and enabling targeted drug delivery through the development of intelligent nanocarriers. As a core technology driving the development of precision medicine, nanomedicine continues to evolve. With ongoing research in nanomaterial engineering and biological interfaces, nanomedicine is expected to play a key role in the development of personalized treatments and intelligent diagnostic and therapeutic systems.

Nanomedicine's transformative potential stems from exploiting the unique size‐dependent properties of 1–100 nm engineered nanomaterials [69, 70]. The nanoscale dimensions endow high specific surface areas, enabling passive accumulation in tumor tissues via the enhanced permeability and retention (EPR) effect while penetrating biological barriers. Targeting ligand (e.g., antibody/peptide) modifications further achieves cell‐specific active targeting, thereby significantly improving therapeutic precision.

The combination of targeting capability and stimulus‐responsive properties enables NPs to precisely release drugs in response to internal/external stimuli, significantly enhancing treatment controllability. Multifunctional platforms simultaneously loaded with imaging agents, drugs, and therapeutic genes improve treatment efficiency. Biocompatibility and degradability ensure safe clearance after functional completion. Controlled drug‐release kinetics enable NPs to adapt to diverse therapeutic requirements, optimizing efficacy while minimizing side effects. Enhanced drug solubility and bioavailability via nanocarriers significantly improve therapeutic outcomes and reduce toxicity.

NPs can be engineered to respond to physiological stimuli such as pH changes or enzyme activities, achieving controlled drug release at diseased sites to minimize damage to healthy tissue. NPs also protect drugs from degradation, prolong their circulation time, and enhance their intratumoral accumulation via EPR. High‐specific surface areas provide efficient loading platforms, while biocompatibility and biodegradability reduce systemic accumulation risks. Collectively, these attributes establish NPs as invaluable tools in drug delivery systems, offering novel strategies for precision medicine.

NPs have been playing an increasingly prominent role in the treatment of neurodegenerative diseases (Table 1), the ability of NPs to penetrate the BBB for targeted drug delivery significantly enhances therapeutic efficacy while reducing systemic side effects. Additionally, nanomaterials improve imaging contrast and treatment precision in magnetic resonance imaging (MRI) and phototherapy [82].

**TABLE 1:** Nanoparticles utilized for research of vascular aging‐related diseases.

Category	Type of nanomaterial	Advantages	Disadvantages	Applications in diseases	References
Inorganic Nanomaterials	Gold Nanomaterials (AuNMs)	Optical, biocompatible, plasmonic features; physical–chemical stability, surface chemistry, multifunctionality	Long‐term toxicity risk, complex synthesis and high cost	AD PD	[71]
	Silver Nanomaterials (AgNMs)	High‐efficiency penetration of the BBB, advantages in endocytic transport	Long‐term retention leading to neurotoxicity, interference with metabolic pathways	VaD AD	[72]
	Mesoporous Silica Nanoparticles (MSNs)	Biocompatible, high drug—loading, sustained—releasing, and easy to functionalize on the surface	Biodegradability and metabolic problems	VaD PD HD	[73]
	Iron Oxide Nanoparticles (IONPs)	Magnetically targetable, multimodal—imaging—compatible, highly biocompatible	Oxidative stress toxicity, iron ion metabolic disorder, difficult large‐scale preparation	VaD, PD, MS	[74]
	Quantum Dots (QD)	High fluorescence intensity, tunable optical properties based on size, surface modifiability	Impact on the environment, high manufacturing cost, high overall toxicity, problems in in vivo clearance	VaD	[75]
Carbon Nanomaterials	Graphene	Conductive for nerve repair, porous for wide drug loading, surface—modified for targeting	Poor biodegradability, long‐term toxicity risk, difficult large‐scale production	VaD AD	[76]
	Carbon Nanotubes (CNTs)	High electrical conductivity promoting nerve regeneration, high mechanical strength, significant drug‐loading capacity	Poor biodegradability, long‐term toxicity risk, strong immunogenicity	VaD, AD, PD	[77]
	Fullerene	Strong antioxidant property, compatibility with multimodal imaging, flexible surface modification	Issues of biodistribution and toxicity	VaD AD	[78]
	Carbon Quantum Dots (CQDs)	High‐sensitivity imaging, drug delivery and controlled release, diversity of surface functionalizatin	Long‐term metabolic risk, poor batch consistency, insufficient photostability	VaD, PD, AD	[79]
Organic Nanomaterials	Polymer Nanoparticles (PNPs)	High biosafety, drug controlled release and targeting, diversity of surface modification	Poor manufacturability, low encapsulation efficiency, long‐term metabolic risk, potential immunogenicity	VaD AD HD	[80]
	Lipid Nanoparticles (LNPs)	Excellent biocompatibility, strong ability to penetrate the BBB, precise drug loading and sustained release	Poor stability, difficult large‐scale production, insufficient long‐term metabolic tracking	VaD AD	[81]

Biocompatibility and safety of nanomaterials have emerged as central research priorities. Certain nanomaterials may exhibit potential immunogenicity or exert inflammatory responses, thereby affecting their in vivo safety and efficacy. Prolonged accumulation could further induce chronic inflammation and other adverse effects.

Nanomaterials tend to accumulate in specific organs (e.g., the brain). Further concerns to be addressed are long‐term toxic damage, potentially induced by non‐degradable materials, and nonspecific distribution leading to drug diffusion in normal tissues, which increases side effect risks. Some materials generate also excessive ROS via oxidative stress responses, disrupting cells and interfering with signaling transduction, gene expression, and protein synthesis.

While penetrating the BBB, various nanomaterials may cause its disruption, increasing inflammation and infection risks. Some metal‐based NPs may release toxic metal ions, damaging the nervous system. In gene therapy, some nanomaterials may interfere with gene expression stability or induce mutations. As drug carriers, the degradation rates of the attached drugs influence their release kinetics, impacting therapeutic efficacy and safety. These caveats need to be considered for clinical applications of drug‐conjugated nanocarriers [83].

## 4 Role of Nanomaterials in the Diagnosis of VaD

By leveraging their unique physicochemical properties, nanomaterials exhibit considerable potential in facilitating the accurate and timely diagnosis of VaD by enabling the precise detection of pathological vascular alterations [33] Among various platforms, polyethylene glycol (PEG)‐coated superparamagnetic iron oxide nanoparticles (SPIONs) have garnered significant attention due to their high biocompatibility and diagnostic versatility [34]. These modified SPIONs efficiently evade phagocytosis by the reticuloendothelial system (RES) and successfully traverse the blood–brain barrier (BBB), providing a robust vehicle for intracranial imaging.

When functionalized with ligands targeting vascular cell adhesion molecule‐1 (VCAM‐1) or other specific endothelial markers, nanomaterials enable high‐contrast imaging of early‐stage vascular inflammation. These targeted strategies facilitate the detection of BBB leakage and microbleeds, which are more indicative of the primary vascular damage underlying VaD‐related cognitive impairment than traditional proteinopathic markers like amyloid‐β (Aβ). In VaD diagnostics, surface‐modified SPIONs utilize MRI to localize these cerebrovascular lesions with high sensitivity [34], offering a diagnostic paradigm that prioritizes vascular etiology over Alzheimer‐related protein aggregates. This advancement is crucial for differentiating VaD from other neurodegenerative disorders and initiating timely therapeutic interventions.

Besides MRI, fluorescence imaging techniques have emerged as effective tools for identifying Aβ deposits through nanostructures [32]. Fluorescent NPs form stable complexes with Aβ via functionalized fluorescent moieties, emitting characteristic fluorescent signals upon excitation to enable precise localization and quantification of Aβ aggregates. In VaD diagnostics, commonly used fluorescent markers include Aβ and vascular endothelial cell‐associated proteins such as vascular cell adhesion molecule‐1 (VCAM‐1) [1].

Optimization of the interaction between nanomaterials and Aβ deposits represents a key research focus in VaD studies. Specifically modified nanomaterials exhibit specific binding to Aβ, while surface engineering strategies incorporating customized moieties can enhance their affinity to improve detection sensitivity and precision.

In summary, nanomaterials are poised to revolutionize the diagnostic paradigm for VaD. Their unique properties and multifunctional capabilities enable the development of highly sensitive and specific assays for detecting Aβ deposits. This advancement is crucial for early diagnosis and timely therapeutic interventions.

## 5 Role of Nanomaterials in the Treatment of VaD

### 5.1 Vascular Damage

VaD is closely associated with vascular injury and BBB dysfunction. Vascular injury instigates an inflammatory response in the vessel wall and the abnormal functioning of vascular endothelial cells. This leads to the disruption of the BBB, resulting in cerebral tissue hypoxia and insufficient nutritional support to brain cells [84]. Since vascular injury is a hallmark of VaD, promoting angiogenesis has become a crucial direction in research focusing on its treatment.

#### 5.1.1 Oxidative Stress and Vascular Aging

Vascular aging is closely linked to oxidative stress, as posited by the free radical theory [85]. With aging, increased ROS production from NADPH oxidases and mitochondria promotes endothelial dysfunction and atherosclerosis [86]. A key feature is the inactivation of nitric oxide (NO) [87], a critical vasodilator, which reduces vascular relaxation and impairs tissue perfusion. Oxidative stress further diminishes NO bioavailability by disrupting endothelial nitric oxide synthase (eNOS) activity, altering substrate/cofactor availability and elevating endothelin‐1 [88]. This imbalance between vasoprotective and vasoconstrictive factors exacerbates coronary dysfunction, myocardial ischemia, and atherosclerosis [89].

Nitrosative stress, driven by peroxynitrite formation from NO and superoxide, directly accelerates vascular aging [90]. Peroxynitrite damages mitochondrial respiratory chains, amplifying ROS production and causing mitochondrial DNA (mtDNA), protein, and lipid damage. Proximity to ROS sources and poor repair mechanisms render mtDNA highly mutation‐prone, reducing energy production and impairing vascular cell function. Mitochondrial dysfunction lowers ATP levels, compromising membrane transport and barrier functions while interacting with oxidative stress to activate NF‐κB and inflammatory pathways [91]. Inflammatory mediators like TNF‐α further stimulate NDPH oxidase, perpetuating oxidative damage. These interconnected mechanisms—oxidative/nitrosative stress, mitochondrial dysfunction, and inflammation—collectively drive vascular aging through a self‐reinforcing cycle of cellular damage and metabolic decline.

#### 5.1.2 Nanotechnology in Vascular Regeneration

Supported by significant breakthroughs of advanced nanotechnology in tackling NO deficits and facilitating neovascularization, the application of nanomaterials in vascular regeneration is becoming increasingly widespread. For example, through the design of specific nanocarriers, angiogenesis‐related factors such as vascular endothelial growth factor (VEGF) and hypoxia‐inducible factor (HIF‐1α) can be effectively delivered, thereby promoting the proliferation and migration of vascular endothelial cells.

The enzyme‐prodrug strategy represents one of the crucial techniques for controllable NO delivery. Taking β‐galactosidase‐catalyzed β‐gal‐NONOate as an example [92], in terms of carrier selection there has been a shift from single‐polymer carriers to composite multifunctional carriers. For instance, the combination of biodegradable polymers with inorganic nanomaterials not only strengthens the stability of the enzyme but also improves the targeting ability of the carrier. Leveraging the unique physicochemical properties of nanomaterials can enhance the efficiency of cell uptake, enabling more precise delivery of NO to the diseased site.

On the other hand, immobilization techniques have been continuously refined, evolving from traditional physical adsorption to precise immobilization methods such as covalent coupling and cross‐linking. This not only reduces enzyme leakage but also maintains high catalytic activity, mitigating the risk of side effects such as fluctuations in heart rate and blood pressure caused by premature release of NO in the plasma [93]. As a result, notable improvements in treatment effects have been observed in applications such as anti‐thrombosis coatings for cardiovascular stents and intraocular pressure reduction in glaucoma treatment [94].

#### 5.1.3 Nanomaterials for NOS and Related Enzymes

The encapsulation of NOS and related enzymes in nanomaterials is a direct approach to achieving in vivo catalytic production of NO. The design of NOS‐containing nanocompartments continues to evolve. Glucose oxidase (GOx) and L‐arginine (L‐Arg) have shown excellent performance in regulating the tumor microenvironment [95]. By optimizing the composition of nanomaterials, for instance, by introducing targeting groups (such as aptamers and antibody fragments) and responsive components (such as pH‐ and redox‐responsive polymers), GOx and L‐Arg can be intelligently released in response to changes in the tumor microenvironment. This process catalyzes NO production and synergistically regulates tumor metabolism, immune escape, and angiogenesis. These modifications have also been expanded to antibacterial applications, where they disrupt bacterial membrane structures and metabolism, as well as to immune regulation, where they modulate immune cell functions and cytokine secretion, enabling multi‐dimensional disease intervention.

#### 5.1.4 Organic Nanomaterials in Angiogenesis

In the domain of organic nanomaterials [96], lipid‐based and polymer NPs have been engineered through chemical modification and compounding strategies. These approaches aim to augment NO loading capacity and precisely regulate the release profile while simultaneously enhancing cell uptake specificity.

With respect to promoting angiogenesis, nanomaterials serve a dual‐purpose function. First, they can act as efficient carriers for delivering angiogenic factors, including proteins and mRNAs [97]. Second, they are capable of emulating the nano‐topological features of the vascular extracellular matrix (ECM). For instance, electrospinning techniques are harnessed to fabricate nanofiber scaffolds that mimic the native ECM. These scaffolds play a pivotal role in facilitating the adhesion, proliferation, and migration of vascular endothelial cells, thereby fostering angiogenesis [98, 99, 100, 101]. Additionally, nanomaterials can modulate gene expression, prominently that of VEGF and HIF‐1α, thus enhancing the angiogenic potential.

Throughout the developmental trajectory, VEGF plays a central and indispensable role in angiogenesis. The interaction between VEGF and its receptor VEGFR‐2 triggers the activation of multiple downstream signal transduction cascades. This activation elicits a series of cellular responses, including the proliferation, migration, and differentiation of endothelial cells, ultimately culminating in the construction of a functional vascular network architecture [102].

**FIGURE 1:**
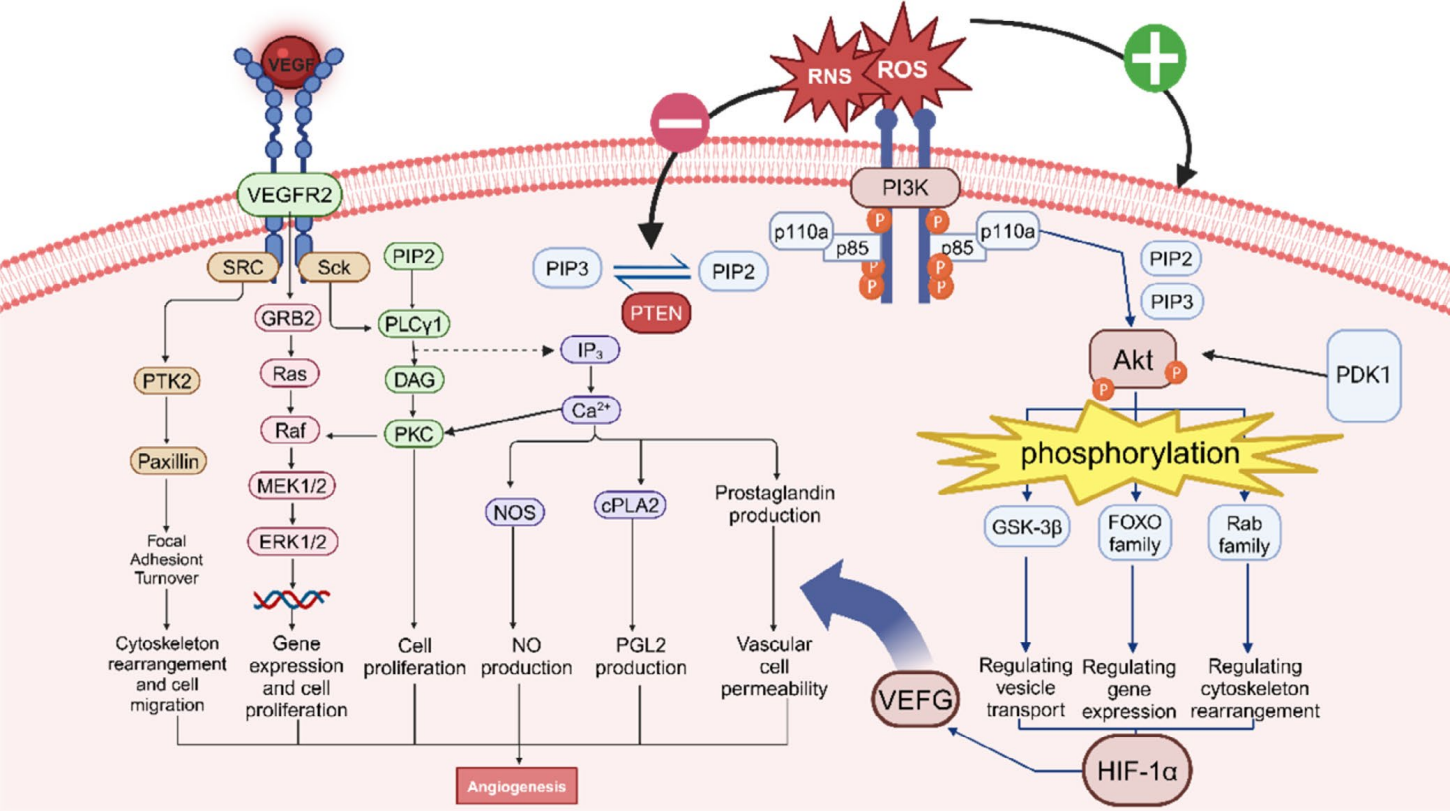
Mechanisms of VEGF/VEGFR2 and PI3K/Akt Pathways in Vascular Dementia. In VaD, the VEGF/VEGFR2 and PI3K/Akt pathways are crucial. VEGF binding to VEGFR2 activates PI3K, converting PIP2 to PIP3, which in turn activates Akt. Akt promotes angiogenesis and inhibits neuronal apoptosis, improving brain blood flow and reducing ischemic damage. This pathway's activation in VaD models decreases brain ischemia and enhances cognitive function, underscoring its importance in VaD pathogenesis and potential therapeutic interventions.

ROS and reactive nitrogen species (RNS) act as pivotal redox signaling mediators intricately involved in the modulation of cell signaling pathways. Despite their well‐known cytotoxic properties, ROS play a crucial role in regulating normal physiological functions. ROS are capable of activating HIF‐1α, which in turn stimulates the release of angiogenic factors such as VEGF [103] (Figure 1). Physiologically optimal concentrations of ROS trigger the activation of transcription factors, leading to upregulation of matrix metalloproteinases (MMPs), which are instrumental in facilitating the migration of endothelial cells. eNOS‐mediated NO synthesis promotes the expression of MMPs and activates the PI3K‐Akt signaling pathway, thereby enhancing endothelial cell migration [104]. Specific nanomaterials, such as zinc oxide NPs and lanthanide NPs, have been shown to activate, contingent upon the presence of ROS and RNS, various kinases of pathogenic relevance to promote endothelial cell migration and neovascularization [105].

Consequently, through the rational design of specific nanoconjugates, it is feasible to fine‐tune the intracellular ROS levels to an optimal range. This modulation not only bolsters the viability of endothelial cells and augments neovascularization but also mitigates oxidative stress‐induced damage. Moreover, the ability of some nanocarriers to stabilize HIF‐1α provides robust support for angiogenesis [106].

### 5.2 Neuronal Loss

**FIGURE 2:**
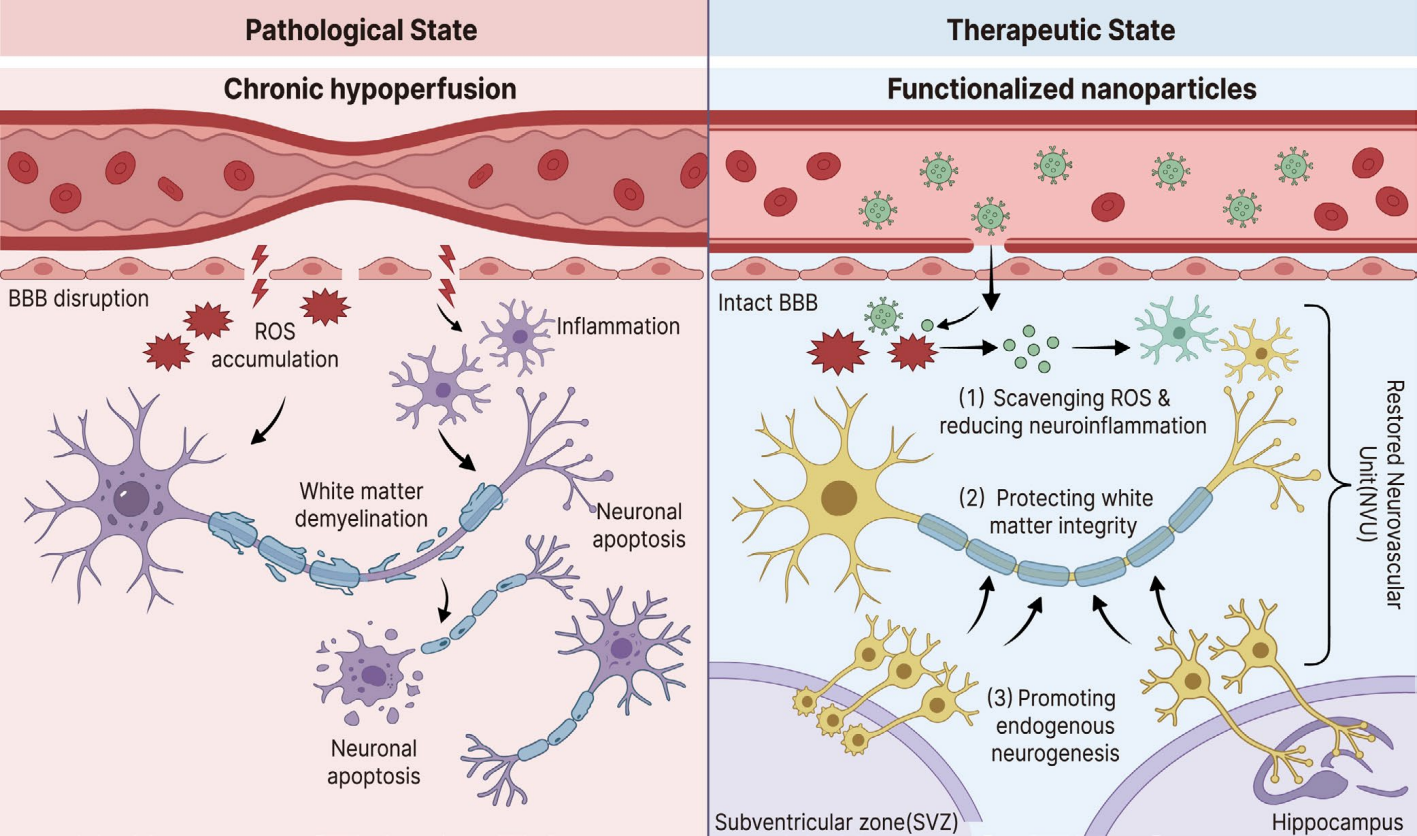
Schematic representation of nanoparticle‐mediated intervention in Vascular Dementia (VaD) pathology. The figure illustrates the transition from vascular injury to neuroprotection. (Left) Pathological State: Chronic hypoperfusion leads to blood–brain barrier (BBB) disruption, oxidative stress (ROS accumulation), and inflammation. The hallmark of VaD is depicted as white matter demyelination and neuronal apoptosis, distinct from amyloid plaque aggregation. (Right) Therapeutic State: Functionalized nanoparticles cross the BBB and target the ischemic penumbra. Mechanisms of action include: (1) Scavenging ROS and reducing neuroinflammation; (2) Protecting white matter integrity by preventing demyelination; and (3) Promoting endogenous neurogenesis in the subventricular zone (SVZ) and hippocampus, ultimately restoring the neurovascular unit (NVU).

Age‐related neuronal attrition and synaptic impairment, as crucial determinants of progressive neurodegeneration and disrupted neural network connectivity, constitute pivotal pathological features in VaD [107]. Neuronal loss, marked by region‐specific depletion through apoptosis or necrosis, arises from multifactorial etiologies including oxidative stress, mitochondrial dysfunction, and neuroinflammation [108]. Concurrently, synaptic dysfunction manifests as neurotransmitter dysregulation, structural synaptic damage, and pathogenic protein accumulation, impairing interneuronal communication and neuroplasticity [109, 110] (Figure 2).

These processes exhibit bidirectional crosstalk: synaptic degeneration precedes neuronal death in early disease stages, while neuronal loss exacerbates network disconnection and disease progression. In Alzheimer's disease (AD), synaptic deficits correlate with memory impairment prior to overt neuronal depletion, highlighting their prognostic significance [110]. Mechanistically, oxidative stress and inflammatory mediators disrupt calcium homeostasis and energy metabolism, accelerating synaptic failure and neuronal apoptosis.

Therapeutic strategies targeting neuroprotection and synaptic preservation are emerging as promising ways to treat VaD [111]. Interventions such as antioxidant agents, anti‐inflammatory therapies, and neurotrophic factor modulation aim to mitigate neurodegenerative cascades [112]. Preclinical studies underscore the potential of dual‐pathway inhibition to synergistically attenuate neuronal loss and synaptic pathology. Elucidating spatiotemporal patterns of these interactions may inform precision therapeutics to delay VaD progression and improve functional outcomes.

#### 5.2.1 CNS Neurogenesis and Nerve Regeneration

Gold NPs (AuNPs) are at the forefront of nanotechnology research aimed at promoting central nervous system (CNS) neurogenesis. In the context of VaD, stimulating the proliferation and differentiation of neural stem cells (NSCs) within endogenously active regions—specifically the subventricular zone (SVZ) and the dentate gyrus of the hippocampus—is a primary strategy for cognitive restoration [113, 114, 115]. Surface modifications of AuNPs endow them with enhanced functionality to specifically interact with NSCs. For instance, biomolecule‐modified AuNPs significantly enhance the survival capacity of neurons in ischemic environments by upregulating the expression of brain‐derived neurotrophic factor (BDNF), cAMP‐responsive element‐binding protein, and stromal cell interaction molecules (STIM1/2) [116, 117, 118].

Moreover, functionalized AuNPs effectively reduce neuroinflammatory responses in these neurogenic niches by suppressing the release of pro‐inflammatory mediators like TNF‐α [119] and regulating the activity of immune cells [120]. By modulating signaling pathways such as nuclear factor‐κB (NF‐κB) [121, 122]., they establish a favorable microenvironment for the integration of newborn neurons into existing circuits.

Carbon‐based nanomaterials, such as graphene oxide (GO) and carbon nanotubes (CNTs), also possess substantial potential for CNS repair [21, 22, 23]. Specifically, graphene scaffolds support the adhesion of neural cells and the outgrowth of neurites, fostering synaptic connectivity within the hippocampal network [123]. CNTs can further promote neuronal communication by establishing nanotube‐nerve hybrid networks [124]. Polymeric nanomaterials [125], exemplified by PLGA NPs [126], act as versatile carriers for delivering neuroprotective agents across the BBB [127]. Instead of targeting amyloid proteins, these carriers are engineered to deliver drugs that mitigate ischemia‐induced neuronal apoptosis and autophagy, thereby accelerating the functional recovery of the neurovascular unit [128].

#### 5.2.2 Protection of the Neurovascular Unit (NVU) and Mitigation of White Matter Lesions

The pathological progression of Vascular Dementia (VaD) is fundamentally driven by the breakdown of the neurovascular unit (NVU) and inflammatory responses triggered by cerebrovascular diseases [129]. Unlike Alzheimer's disease, which is characterized by extracellular protein aggregation, VaD involves region‐specific neuronal depletion through apoptosis or necrosis resulting from chronic hypoperfusion and oxidative stress [130]. Imaging features of small vessel disease (SVD), a primary cause of VaD, include lacunes and white matter lesions that signify deep structural damage to the brain's neural connectivity [131, 132].

Nanomedicine offers a strategic shift toward protecting the NVU and mitigating these ischemic injuries. Nanomaterials, such as polymeric nanoparticles (PNPs) and specifically surface‐modified PLGA nanoparticles, serve as efficient drug carriers capable of crossing the blood–brain barrier (BBB) to deliver neuroprotective agents directly to the ischemic penumbra [133]. These nanoplatforms can safeguard nerve cells from the toxic impact of oxidative stress and inflammatory mediators that disrupt calcium homeostasis and energy metabolism [134, 135].

Furthermore, the application of nanomaterials shows significant potential in reducing vascular damage and decreasing neuronal loss [136]. By utilizing targeted nanoconjugates to modulate intracellular reactive oxygen species (ROS) levels, it is possible to bolster the viability of both endothelial cells and neurons while mitigating oxidative damage. These strategies focus on preserving white matter integrity and stabilizing disrupted neural networks, directly addressing the core pathophysiological mechanisms that lead to cognitive decline in VaD patients [137].

### 5.3 Neuroinflammation

#### 5.3.1 Neuroinflammatory Mechanisms in VaD

In the context of cerebral ischemia/reperfusion injury [138], neuroinflammation plays a complex and crucial role. Microglia, the resident immune cells of the CNS, respond to damage‐associated molecular patterns and inflammatory mediators released after cerebral ischemia by altering their morphology and gene expression patterns, thereby activating the NF‐κB pathway [139].

Microglial cells can assume different phenotypes. M1‐type microglia are activated in response to pathogen invasion, tissue damage, or inflammatory signals, releasing pro‐inflammatory cytokines and chemokines including TNF‐α, IL‐6, IL‐1β, IL‐12, and CCL2 [140, 141]. Moreover, M1‐type microglia express NADPH oxidase, inducible nitric oxide synthase (iNOS), major histocompatibility complex II (MHCII), as well as various integrins and costimulatory molecules. These factors collectively contribute to neural damage and further enhance tissue destruction [141, 142, 143]. In contrast, M2‐type microglia exhibit anti‐inflammatory properties [144]. They are induced by anti‐inflammatory cytokines such as IL‐4 [145] and IL‐13, and produce anti‐inflammatory cytokines, including IL‐10 and TGF‐β [146, 147, 148], as well as growth factors and neurotrophic factors such as insulin‐like growth factor 1 (IGF‐1), fibroblast growth factor, and BDNF [149]. M2‐type microglia support the reconstruction of the ECM and tissue repair and maintain neuronal survival by promoting the phagocytosis of cell debris and misfolded proteins. Results from experimental models of autoimmune encephalomyelitis demonstrate that the polarization shift of microglia from M1‐type to M2‐type is crucial for the treatment of neurodegenerative diseases [150].

**FIGURE 3:**
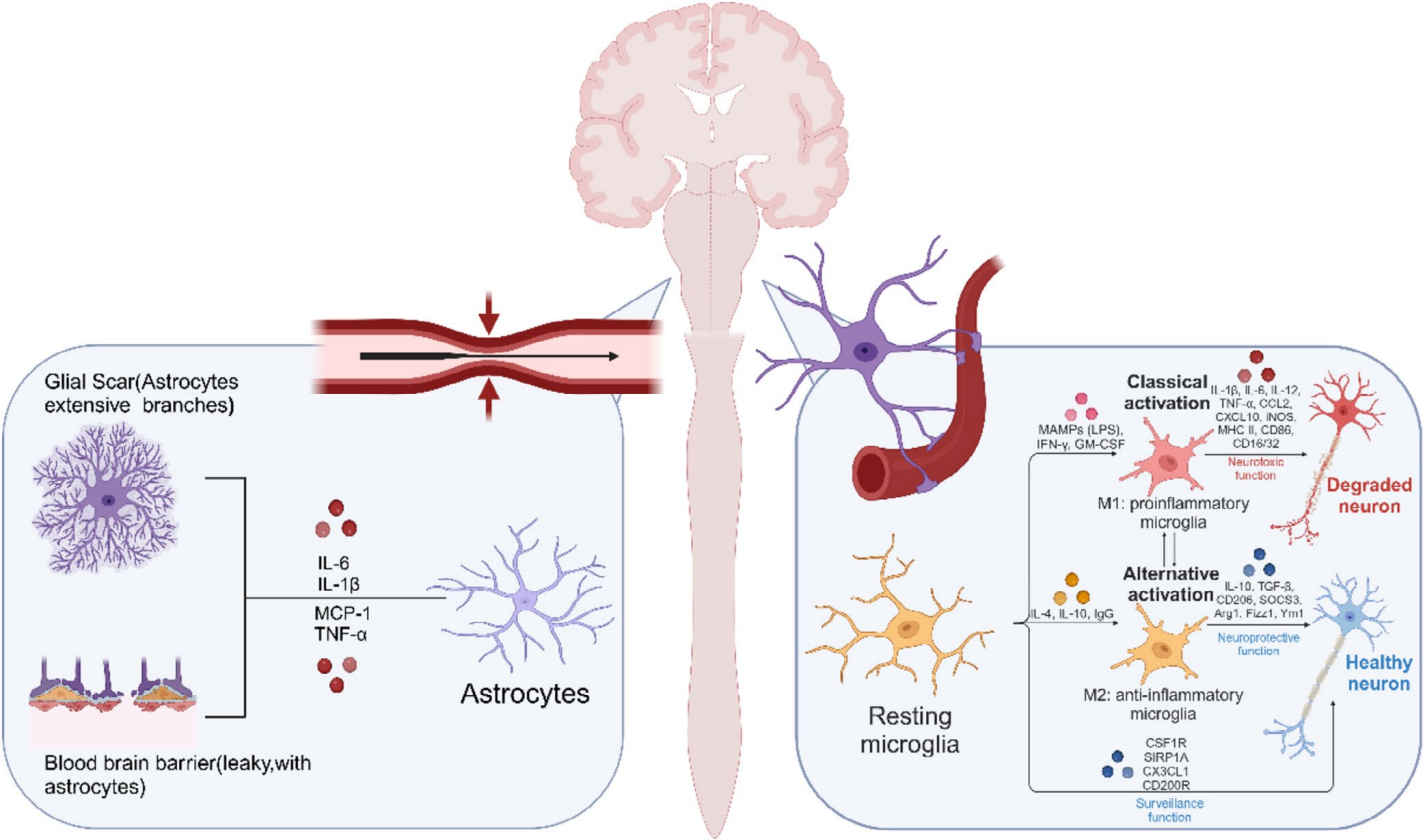
The crucial roles of astrocytes and microglia in the central nervous system. In the left‐hand part, astrocytes form extensive branches to constitute glial scars. Meanwhile, their influence renders the blood‐brain barrier leaky. Moreover, astrocytes release inflammatory factors such as IL‐6, IL‐1β, and MCP‐1. The right‐hand side demonstrates the immune response process. There are two pathways, namely classical activation and alternative activation. The former is associated with M1 pro‐inflammatory macrophages, while the latter involves M2 macrophages. In addition, resting monocytes and various cytokines and markers in different activation states are also included.

Astrocytes are activated after cerebral ischemia, leading to the formation of glial scars and the disruption of the BBB [151]. They participate in the inflammatory response by releasing inflammatory factors such as IL‐1β and monocyte chemoattractant protein‐1 (MCP‐1) [152]. TNF‐α, IL‐6, and IL‐1β play important roles in the neuroinflammatory process [153]. These inflammatory factors can affect cognitive function and are closely related to the development of VaD(Figure 3).

#### 5.3.2 ROS‐Targeted Nanomaterials for Multi‐Pathway Intervention in VaD

Nanozymes [154] that mimic the enzymatic activities of superoxide dismutase (SOD) and catalase (CAT) promote the scavenging of ROS through catalytic reactions [155, 156, 157]. The structure–activity relationship model of cerium oxide (CeO_2_) nanomaterials provides a theoretical basis for the design of efficient nanozymes [158]. CeO_2_ nanomaterials can effectively scavenge superoxide anions and hydrogen peroxide due to the reversible conversion of Ce(III)/Ce(IV). In addition, the oxygen vacancies on the surface of CeO_2_ nanomaterials are crucial for catalytic activity [159], and their concentration and distribution affect the catalytic efficiency of the materials. Although CeO_2_ also exhibits a non‐catalytic chemical reduction pathway, this irreversibly alters the oxygen vacancies on the surface of the NPs, leading to a decline in nanozyme activity. In summary, CeO_2_ and CeO_2_‐based nanomaterials effectively scavenge excessive ROS by mimicking the activities of SOD and CAT, providing a new strategy for the treatment of oxidative stress‐related diseases.

Redox‐active ROS‐scavenging NPs scavenge free radicals through redox reactions. For example, in curcumin‐based NPs [160], curcumin's phenolic hydroxyl groups are critical for their ROS‐scavenging activity. These groups have a low O‐H bond dissociation energy and can easily donate hydrogen atoms to free radicals; this converts them into more stable molecules, effectively reducing oxidative stress [161]. Due to their high surface‐to‐volume ratio and improved bioavailability, these NPs show great potential in the treatment of conditions associated with oxidative stress such as neurodegenerative diseases, cardiovascular diseases, and cancer. However, further studies assessing their safety and biocompatibility are needed to ensure their reliability in clinical applications. ROS‐responsive polymer nanomaterials contain functional groups that change physical properties or chemical bond breakage under the action of ROS [162], reducing ROS levels. In addition, other types of nanomaterials, such as melanin‐based [163], polydopamine‐based [164], and selenium‐based [165] NPs were shown to scavenge ROS through direct chemical reduction reactions. In turn, carbon nanomaterials directly scavenge ROS by disrupting their structures through electron transfer. The targeted scavenging strategy [166] enables nanomaterials to specifically act on organelles (mitochondria) or diseased sites to reduce oxidative damage. In combination therapy, nanomaterials are paired with drugs to scavenge ROS and deliver drug treatment. For example, selenium NPs were used to transport neuroprotective drugs for the treatment of spinal cord injury [167].

Free‐radical‐trapping NPs are nanoscale materials specifically designed to neutralize excessive ROS and RNS. They may be highly useful in the treatment of oxidative stress‐related diseases by capturing and scavenging these highly reactive free radicals. These NPs work through different mechanisms. TEMPO‐based NPs [168] carry stable nitroxide radicals that capture unpaired electrons. Through reversible single‐electron redox reactions, the oxidized nitroxide is converted into hydroxylamine [169], providing antioxidant functions. TEMPO can penetrate cell membranes, scavenge superoxide anions and peroxides, and reduce the Fenton reaction and the recombination between free radicals [169]. Fullerene NPs [170] are excellent electron acceptors due to their conjugated double‐bond structure and capture ROS by accepting their unpaired electrons. The design and synthesis of water‐soluble fullerene derivatives further enhance their ROS‐scavenging ability and enable them to target specific cells and tissues [171]. Prussian blue NPs scavenge various types of free radicals, including superoxide anions, hydroxyl radicals, hydrogen peroxide, and organic peroxides by mimicking the activities of enzymes such as peroxidase (POD), CAT, and SOD [172].

The various NPs described above show great therapeutic potential in the treatment of inflammatory, neurodegenerative, and cardiovascular diseases due to their high antioxidant capacity. However, their clinical applications still face many challenges, including ensuring the biocompatibility, targeting, and long‐term stability of NPs. Further in‐depth studies are also required to evaluate specific mechanisms of action and potential long‐term toxicity in vivo. Future research should thus focus on optimizing the design of NPs, improving their therapeutic efficiency, and evaluating their safety and effectiveness in clinical applications.

## 6 Nanotechnology‐Driven Strategies for VaD: From Bench to Bedside

Advances in nanotechnology have allowed the development of drug nanocarriers with specific targeting, controlled release characteristics, and excellent biocompatibility, aiming to break through the BBB and achieve precise delivery of drugs to damaged brain regions. For example, solid‐lipid‐NPs (SLNPs) have been formulated to carry donepezil, a reversible, non‐competitive inhibitor of acetyl‐cholinesterase approved by the FDA for the treatment of mild to moderate dementia related to AD. When administered via the nasal mucosa, these NPs can effectively cross the BBB, deliver the drug to the brain, and improve cognitive function [173]. Yasir et al. improved the administration efficiency by optimizing the SLNP formulation [174].

As natural nanocarriers, cell‐derived exosomes are highly effective in transporting therapeutic proteins and RNAs to alleviate neurodegenerative lesions in VaD. For instance, targeted exosomes modified with rabies virus glycoprotein can specifically deliver glyceraldehyde‐3‐phosphate dehydrogenase small interfering RNA (GAPDH siRNA) into neurons, microglia, and oligodendrocytes, significantly reducing the level of β‐site amyloid precursor protein cleaving enzyme 1 (BACE1), one of the key targets for the treatment of neurodegenerative diseases [175, 176]. Jiang et al. developed nanogels formed by hyaluronic acid modified with epigallocatechin‐3‐gallate (EGCG) and curcumin for the treatment of AD. The NGs exhibited an inhibitory efficiency of over 50% against Aβ, and the cell survival rate was significantly increased after administration of bi‐modified, versus mono‐modified, NGs [177]. In a stroke model (which is often used to replicate the pathological conditions associated with VaD), the treatment with liposome‐encapsulated drug (e.g., C‐lipo/CA, TanIIA‐PLNs) was shown to effectively reduce the degree of brain injury and promote functional recovery [178, 179].

Despite remarkable progress in the field of nanotechnology within the biomedical domain, with demonstrated potential in combating major diseases such as cancer and cardiovascular disease, the number of clinical trials on nanomaterials specifically for VaD remains relatively limited. Currently, drug therapy remains the primary clinical intervention for VaD. However, traditional drugs often struggle to effectively cross the blood–brain barrier, significantly undermining treatment efficacy. Nanomaterials, characterized by their size and surface properties, show promise in enhancing drug delivery to the brain and offer therapeutic hope for VaD. However, clinical research in this area remains limited, with only a few trials focusing on their application in VaD treatment. Additionally, some research has explored the combined application of nanomaterials and cell therapies, and preliminary findings have verified the synergistic effects of this combined strategy. However, most of these studies are at an early stage, and there is still a long way to go before they can be translated into clinical practice.

In view of this, more researchers should conduct clinical studies on nanomaterial–based VaD treatment. Future trials should be multi–center and large–scale to expand sample sizes, improving result reliability. Mechanisms of nanomaterials also require further exploration. Advanced imaging and biomarkers can monitor their in–vivo distribution and metabolism in real–time, facilitating precision medicine. Standardized evaluation systems are also needed to objectively compare the efficacy of different nanomaterials and treatment strategies. Through these efforts, we can expect significant progress in this field, bringing new treatment options to VaD patients.

## 7 Future Challenges in the Application of Nanomaterials for VaD Treatment

Despite significant promise, the application of nanomaterials in VaD diagnosis and treatment faces several hurdles. The long‐term neurotoxicity and in vivo metabolism of many nanomaterials remain undetermined. Additionally, the lack of a systematic biocompatibility assessment framework severely hinders clinical translation. In the complex pathological milieu, protein coronas (formed by the binding of serum proteins to NPs) may disrupt targeted modification strategies, decreasing targeting precision and compromising BBB penetration efficiency. VaD pathology involves interactions among damaged vascular cells, neuroinflammation, and oxidative stress. However, most research focuses on single pathways rather than comprehensive multi‐target strategies. Recent advancements in nanorobotic technology have revolutionized precision medicine [180]. While the BBB plays a crucial role in protecting the CNS from xenobiotics, it remains a major obstacle to drug delivery. For many NPs, BBB penetration efficiency still requires optimization. Multifaceted approaches have been developed to enhance BBB permeability, including osmotic shock, sonoporation, cell‐penetrating peptides, magnetic gradient‐enhanced transport of magnetic NPs, and focused ultrasound (FUS) [181]. Current BBB‐penetrant nanomaterials encompass polymers (e.g., PLGA, PLA, and PLBA), AuNPs, liposomes, micelles, dendrimers, exosomes, and single‐domain antibodies (nanobodies). Among these, nanorobots(e.g., Self—propelled nanorobots, External field—propelled nanorobots and Motile microorganism—propelled biohybrid nanorobots)with the abilities of precise targeted transport and controlled drug release, as well as good biocompatibility and safety emerge as the most promising targeted drug carriers [182].

A CNS‐traceable delivery system reported by Huang et al. [183] leverages neural stem cell membranes to enhance BBB traversal and achieve spatiotemporal targeting of neurons. This system protects payloads from enzymatic degradation and accumulates preferentially at lesion sites, thereby augmenting therapeutic indices. Surface‐functionalized nanorobots integrate active targeting via ligand conjugation, microenvironment‐responsive targeting, and imaging‐guided navigation. Programmable drug release is achieved through stimuli‐responsive mechanisms, enabling spatiotemporally controlled therapy. These microdevices navigate the vasculature to precisely localize ischemic foci, delivering thrombolytics or neuroprotectants while minimizing off‐target effects. Post‐intervention clearance via endocytosis [184] further reduces cytotoxicity.

Nanovaccines represent an innovative immunomodulatory platform [185], priming adaptive immunity against pathological mediators to prevent cerebrovascular injury and chronic neuroinflammation. Prophylactic nanotechnology interventions, deploying antioxidant‐ or anti‐inflammatory‐loaded NPs, aim to pre‐emptively arrest cerebrovascular degeneration by neutralizing oxidative stress and inflammatory cascades. These strategies collectively advance the translational potential of nanomedicine in VaD management.

## 8 Conclusion and Prospect

This review explores the cutting‐edge applications of nanomaterials in VaD diagnosis and treatment, with a focus on targeted drug delivery, diagnostic innovations, and pathological mechanism intervention. Nanocarriers like liposomes, PLGA NPs, and exosomes can penetrate the BBB to precisely deliver drugs (e.g., donepezil, siRNAs), increasing drug concentrations in damaged brain areas and improving cognitive function. Diagnostic tools such as SPIONs and QDs facilitate the detection of Aβ deposits in cerebral blood vessels for early VaD diagnosis. NP‐mediated delivery of pro‐angiogenic factors (VEGF, HIF‐1α) enhances blood perfusion, while functionalized AuNPs inhibit Aβ aggregation. Additionally, engineered polymer nanomaterials promote synapse regeneration, nanozymes (CeO₂) scavenge ROS, and modified dendritic polymers polarize microglia to the M2 type to reduce neuroinflammation.

This account describes the use of nanomaterials to target multiple aspects of VaD pathology (BBB damage, oxidative stress, inflammation), providing an innovative treatment framework. It also highlights the ability of diverse nanostructures to overcome the issue of BBB penetrance in drug delivery, and their potential to advance personalized precision medicine, as evidenced by effective brain targeting via nasal‐delivered SLNPs. However, the long‐term neurotoxicity of some nanomaterials is unclear; targeting strategies need improvement, and most data are from animal studies.

Future research should continue to develop intelligent, biocompatible nanocarriers with controlled release kinetics. Combining AI‐assisted design, organoid models, and single‐cell sequencing would help analyze nanomaterial‐neural microenvironment interactions. Promoting emerging technologies (nanovaccines, nanorobots), establishing safety evaluation methods, and exploring synergies with photothermal therapy and gene editing for diagnosis‐treatment‐monitoring integration are crucial steps to breaking VaD treatment bottlenecks and improving patient outcomes.

## Author Contributions

The initial idea for this review was conceived by X.L., J.H., X.Z., and X.D. The manuscript was written and revised by X.L. and J.H. In addition, J.H. and C.S. contributed to the preparation of the illustrations. Z.L., Y.H., and H.W. edited the manuscript. X.L., X.Z., and X.D. read, reviewed, and approved the final manuscript. The final version of the manuscript was approved by all the authors.

## Funding

This study was supported partially by the Provincial Natural Science Foundation of Jiangxi Province (Grant 20224BAB206040 to X.Z.), the Science and Technology Project Founded by the Education Department of Jiangxi Province (Grant GJJ2401808 to H.W.), Foundation of Students’ Platform for Innovation and Entrepreneurship Training Program (Grants 202411843024 to X.Z. and S202411843050 to C.S.), Jiangxi Province Culture and Art Science Planning Project (Grant YG2022188 to X.D.), and Jiangxi Province Humanities and Social Sciences Research Project (Grant JC22124 to X.D.).

## Disclosure

Declaration of generative AI in scientific writing: During the preparation of this work, the author(s) did not use any generative AI or AI‐assisted technologies. The entire manuscript was created, researched, written, and revised by the author(s) in the traditional way. This included searching through academic databases by hand, forming research questions and hypotheses based on the authors' knowledge, and collecting and analyzing data using standard scientific methods. Every sentence, paragraph, and section was written by the author(s) to make sure it was original, accurate, and honest. The author(s) take full responsibility for the content of the published article. They checked every part of its creation carefully. They did not use automated text generation or AI help beyond basic tools like grammar and spell checkers. These tools are used to make the text easier to read but do not add to the main ideas or knowledge of the writing.

## Ethics Statement

This study does not involve any human subjects or animal experiments, so there is no need to obtain consent forms.

## Consent

All authors have read and agreed to the final version of this manuscript and consent to its submission to your journal for publication. We confirm that all authors are aware of and agree to the content and publication of the manuscript.

## Conflicts of Interest

The authors declare no conflicts of interest.

## Data Availability

This study has no new experimental data. The data in this review come from studies published before. These studies are listed in the References section.
